# Eating Behaviors and Energy and Nutrient Intakes in Children with Autism Spectrum Disorder with and Without Sensory Integration Difficulties

**DOI:** 10.3390/children13040478

**Published:** 2026-03-30

**Authors:** Gözde Ede İleri, Yeliz Serin, Pelin Akın, Yusuf Ataş, Sude Çınar

**Affiliations:** 1 Department of Nutrition and Dietetics, Faculty of Health Sciences, Çankırı Karatekin University, Çankırı 18100, Türkiye; yyusuffatass@gmail.com (Y.A.); acelyasude.5@gmail.com (S.Ç.); 2 Department of Nutrition and Dietetics, Faculty of Health Sciences, Çukurova University, Adana 01330, Türkiye; yserin@cu.edu.tr; 3 Department of Statistics, Faculty of Science, Çankırı Karatekin University, Çankırı 18100, Türkiye; pelinakin@karatekin.edu.tr

**Keywords:** autism spectrum disorder, sensory integration dysfunction, children, eating behavior, nutritional status

## Abstract

**Highlights:**

**What are the main findings?**

- The higher frequency of skipping main meals and the significantly lower intake of energy and many essential nutrients in the ASD + SID group indicate that these children are at higher nutritional risk.
- Identification of high scores on the drinking passion subscale as an independent risk factor for SID highlights the importance of incorporating sensory-based approaches into nutritional assessments.

**What are the implications of the main findings?**

- Sensory processing disorder accompanying ASD negatively affects children’s eating patterns, significantly reducing their energy and nutrient intake.
- Sensory processing disorder increases the risk of energy and nutrient deficiency, revealing that sensory factors are crucial but have been ignored in nutritional assessments and interventions in children with ASD.

**Abstract:**

**Background/Objectives**: Sensory processing disorders (SID) are common in children with autism spectrum disorder (ASD) and can influence children’s eating behaviors. Evaluating the nutritional status of children with ASD is crucial for families or caregivers to manage their feeding. Therefore, this study aimed to compare the eating behaviors and dietary intake between children with ASD and children with ASD + SID. **Methods**: This cross-sectional study included 72 children with ASD aged 6–15 years, of whom 36 also had SID. Sociodemographic information and dietary habits of children were collected. The children’s body weight and height were measured. Children’s eating behaviors were assessed using the Children’s Eating Behavior Scale. Dietary intake was obtained using 3-day food consumption records. **Results**: The rate of overweight was higher in children with ASD compared to children with ASD + SID, but there was no statistically significant difference between the groups (*p* > 0.05). Children with ASD + SID were more likely to skip main meals than children with ASD (*p* < 0.05). Children with ASD + SID had significantly lower dietary reference intake levels of energy, macronutrients, fiber, PUFAs, vitamin E, B1, B6, folate, potassium, calcium, magnesium, phosphorus, and iron compared to children with ASD (*p* < 0.05). Increased scores on the drinking passion subscale were identified as a risk factor for SID (OR = 2.15, 95% CI [1.30, 4.30], *p* = 0.005). **Conclusions**: The higher frequency of skipping main meals, significantly lower energy and nutrient intake in the ASD + SID group indicates that these children are at higher nutritional risk. Incorporating sensory-based assessments and interventions into nutritional management may be crucial.

## 1. Introduction

Autism spectrum disorder (ASD) is a neurodevelopmental condition that begins in early childhood and is characterized by restricted, repetitive behaviors and deficits in verbal and nonverbal communication and in interests [1]. The Centers for Disease Control and Prevention (CDC) data in the US show that 1 in every 88 children has ASD, and the risk of ASD is approximately 5 times higher in boys than in girls [2]. Globally, the World Health Organization (WHO) reported in 2021 that approximately 1/127 individuals (or, alternatively, approximately 1/100 children) have ASD [3]. Within the scope of the ASD screening program conducted in Türkiye, the Modified Checklist for Autism in Toddlers (MCHAT) was administered to 44,045 children, and 10.5% (*n* = 4605) were reported as screening-positive [4].

Children with ASD experience difficulties in social interaction and communication, as well as delays in behavioral, linguistic, and cognitive development; the disorder persists throughout their lives. The symptoms can vary from person to person in terms of appearance and severity with age and maturation [5,6]. Deficits in communication and social interaction, sensory processing difficulties, repetitive and stereotyped behaviors, and problems involving language and speech seen in children diagnosed with ASD also lead to various behavioral difficulties. Examples of these behaviors include feeding problems, abnormal sleep behaviors, self-stimulatory behaviors, temper tantrums, aggressive behaviors, fears and phobias, hyperactivity, obsessions, motivational and attentional problems, and teeth grinding [7,8].

Feeding behaviors in children with ASD have become an important area of research due to their close relationship with growth and developmental outcomes. More than half of these children exhibit food selectivity, aversion to certain foods, and sensory sensitivities related to taste, smell, and texture; these characteristics reduce dietary diversity and can lead to impaired micronutrient adequacy [9,10]. Mealtime behavioral difficulties are also more pronounced compared to typically developing peers, with differences focused more on variety and adequacy than on increased total energy intake [11]. Nutritional interventions may play a role in managing gastrointestinal problems and some behavioral difficulties in children with ASD [12,13,14]. However, because much of the current literature originates from Western countries, data on specific feeding patterns of children with ASD in Türkiye remain limited.

Excessive consumption of energy-dense foods high in carbohydrates and fats increases the risk of obesity, while also leading to deficiencies in energy, protein, and nutrients in children with ASD [15,16,17,18]. Children with ASD reject many foods, require special eating utensils and specialized food preparation, exhibit very narrow food preferences, and display unique nutritional behaviors. Nutrition, an aspect of daily living, can be adversely affected by sensory changes. Eating problems such as refusing solid foods, not chewing, selective eating, and consuming inedible substances are also common in children with ASD [19,20,21]. It is challenging to diversify the diets of children with ASD, who are resistant to novel foods. Nutrient deficiencies (lipid-soluble vitamins, group B vitamins, vitamin C, iron, zinc, and magnesium) can be seen in children with ASD because of extreme food selectivity; these children are often highly sensitive to the texture of foods and drinks. While such feeding problems disappear with age in normally developing children, they persist or worsen in children diagnosed with ASD [22,23,24].

Several explanations have been proposed for the feeding difficulties observed in children with ASD. Some of these views include gastrointestinal problems, sensory integration problems, restrictive and repetitive behavior patterns, and insistence on sameness. While these conditions sometimes cause feeding problems individually, they can also occur simultaneously [25,26]. A higher incidence of gastrointestinal symptoms has been reported in children with ASD. It has been suggested that gastrointestinal symptoms and food selectivity may lead to inadequate food intake and, consequently, to abnormal anthropometric measurements [27]. In a study of children with ASD, 84.8% exhibited food selectivity, and the degree of ASD was associated with the consumption of milk, yogurt, oilseeds, rice/pasta, and fruit (*p* < 0.05). It was also reported that there was a significant association between ASD severity and the frequency of consumption of eggs, legumes, and other grains [28].

Sensory processing is related to how individuals interpret and organize input from various sensory systems, including auditory, visual, taste and smell, somatosensory, vestibular, and intra-body signals [29]. Sensory differences reflect global, multivariate conditions and are reported in up to 90% of individuals [30]. The DSM-5 states that sensory processing difficulties are considered a general set of defining characteristics encompassing hypersensitivity or hyposensitivity to sensory stimuli, as well as sensory stimulus-seeking behaviors [31]. Children with ASD are considered a vulnerable group because hyper- and hyposensitivity affect sensory integration. Sensory differences are associated with ASD not only as basic symptoms but also in their effects on various behavioral and clinical features [32]. The eating experience relies on multiple sensory modalities, including the visual appearance of food, eating-related auditory cues, gustatory and olfactory perception, and tactile inputs arising from textural features. The sensory processing differences (hyper-sensitivity and hyposensitivity) and impairments in multisensory integration observed in autism spectrum disorder can lead to atypical subjective processing of external sensory input, thereby altering eating behaviors. Selective eating and limited food variety are common in children with ASD; systematic reviews report that these difficulties are consistently associated with impaired sensory processing/perception and often show a positive correlation with rigidity and defiant behavior [33,34].

To conduct educational interventions, it is important to determine the relationship between feeding patterns and eating behaviors in children with ASD and sensory integration disorder. Previous studies have shown that differences in sensory processing have significant effects on both behavioral patterns and daily living habits in children with ASD. These studies indicate that ASD is characterized by atypical sensory processing that affects spatial and temporal perception; therefore, sensory processing mechanisms in the visual and auditory modalities have been examined in detail. Sensory hypersensitivity has been reported to affect food selectivity and eating behaviors across cultures [35,36,37,38]. Although nutritional status and eating behaviors have clinically significant effects on children with ASD, especially those with sensory integration disorder, studies that comparatively examine these aspects in children with and without SID are limited. Overall, the literature indicates that differences in sensory processing affect the behavioral and feeding patterns of children with ASD, yet the full extent of these interactions remains poorly understood. Therefore, this study aims to fill this literature gap by comparatively evaluating the feeding patterns and eating behaviors of children with ASD, stratified by the presence or absence of sensory integration disorder. We hypothesized that sensory integration impairment would affect feeding patterns and eating behaviors in children with ASD.

## 2. Materials and Methods

### 2.1. Study Design and Participants

This single-center cross-sectional study was conducted at a special education and rehabilitation center in İstanbul that serves children with special needs. This center provides individualized education, sensory integration therapy, and behavioral support programs for children diagnosed with ASD, and follows up with participants as part of routine assessment processes. Throughout the study, assessments were carried out in the center’s quiet assessment rooms, free from distracting stimuli. All data-collection processes were conducted in the presence of specialists working at the center and under standard practice conditions. Inclusion criteria were a diagnosis of ASD or SID by a pediatrician in accordance with the DSM-V criteria [1], and age 6–15 years. In this study, no additional standardized tool was used to reconfirm diagnoses. During each child’s admission to a participating institution, diagnoses were made and confirmed by the relevant specialist physician (pediatrician), based on DSM-5 criteria, as part of routine clinical procedures. Similarly, for the exposure variable of the study, SID, our research team did not apply an independent diagnostic tool; instead, this condition was classified based on the clinical diagnosis made by the relevant physician. In this study, children were excluded to reduce confounding factors for the following reasons: (i) chronic, metabolic, and genetic disorders requiring personalized/therapeutic diets disrupt typical eating patterns and reduce comparability; (ii) specific diets, such as gluten-free/casein-free diets, can have independent effects on behavioral and nutritional outcomes in ASD; (iii) nutritional supplements and medications that affect choline metabolism can alter natural intake patterns and related biomarkers; (iv) psychotropic medications (stimulants may reduce appetite; atypical antipsychotics may increase appetite/weight) systematically distort energy and nutrient intake estimates; and (v) the intakes of individuals not receiving oral nutrition could not be methodologically compared with clinically prescribed and unrestricted home eating behaviors.

Since there was insufficient prior data on food selectivity and sensory hypersensitivity in the ASD sample, reference values for a physiological parameter with a well-defined normative distribution and consistent population variability were used to calculate the sample size. The aim of this approach is to estimate only the minimum required number of participants in a conservative, standardized manner; the selection of the parameter is not intended to establish a conceptual or functional link with the study variables, but rather to ensure methodological robustness and applicability.

The sample size for the study was calculated using the Power Analysis and Sample Size (PASS) 2026 package, with a type I error level of α = 0.05, a type II error level of β = 0.20, and a power of 1 − β = 0.80. Accordingly, at least 36 children diagnosed with both ASD and SID who met the inclusion criteria for this study, and at least 36 age-matched children with ASD-only, were needed to detect a significant difference.

The study was approved by the Health Sciences Scientific Research and Publication Ethics Board of Çankırı Karatekin University (Approval No: 10, Date: 10 November 2024) and was conducted in accordance with the Declaration of Helsinki. Additionally, this article was prepared in accordance with the STROBE checklist.

### 2.2. Data Collection

Data were collected between January and May 2024 using a questionnaire developed by the researchers. Children were screened for eligibility based on inclusion and exclusion criteria established using the center’s registered pool. Mothers of eligible children were informed face-to-face about the purpose and scope of the study; participation was entirely voluntary. The mothers answered the questions, and the researchers recorded the responses. Before data collection, the researchers informed the mothers about the study’s purpose and content. Mothers who volunteered to participate in the study signed a parental consent form that included detailed information about the study’s purpose, the measurements, and the data to be collected. Assessments were conducted in standard assessment rooms located within the center.

We used a five-part questionnaire to collect data from mothers. The first part of the questionnaire includes questions about the children’s sociodemographic characteristics, such as age, gender, birth order, family history of ASD, and parental consanguinity. The second part of the questionnaire includes questions about dietary habits (number of main meals, skipping meals). In the third section of the questionnaire, the researchers measured and recorded body weight and height. The fourth part of the questionnaire includes the Child Eating Behavior Questionnaire. The fifth part of the questionnaire includes 3-day food consumption data. The sections containing demographic and clinical characteristics were structured according to standardized data-collection frameworks used in previous similar pediatric nutrition and ASD studies. However, the questionnaire is not a scale but a descriptive data-collection form created to systematically collect participant characteristics and therefore does not have a psychometric structure that would require a separate validity and reliability study.

### 2.3. Anthropometric Measurements

Body weight was measured using a scale with 0.01 kg precision after overnight fasting and after their first morning void. We measured the height of children using a Seca portable anthropometric device, with feet together and the head positioned in the Frankfurt plane, according to the standard procedure [39]. We calculated the body mass index (BMI) as weight (kg)/height (m^2^). We evaluated body weight, height, and BMI based on the World Health Organization classification using growth charts [40]. Accordingly, we considered children with a body weight for age ≥95th percentile as obese, those between the 85th and 95th percentiles as overweight, those between the 15th and 85th percentiles as normal, and those <5th percentiles as underweight. Additionally, weight-for-age (WA), height-for-age (HA), and BMI-for-age (BAZ) z-scores were calculated. BAZ < −2 and WAZ < −2 were classified as underweight; BAZ 1–2 indicated overweight; and BAZ > 3 indicated obesity. Children’s height was considered normal if it was between −1 SD and +1 SD, short (stunted) if <−2 SD or <3rd percentile, very short (severely stunted) if <−3 SD, and tall if >+2 SD or >97th percentile.

### 2.4. Dietary Assessment

We assessed children’s dietary intake using a 3-day food record that was designed to include two weekdays (Thursday and Friday) and one weekend day (Saturday or Sunday), so that mothers could report their children’s usual eating habits at home. Families were given written and verbal instructions on how to record in detail all foods and beverages consumed each day, including quantity and preparation method. We used the Food and Nutrition Photo Catalog Measurements and Amounts, as well as food models, to determine the portion size and help parents estimate the children’s daily food consumption [41]. We analyzed dietary intake using the Computerized Nutrient Analysis Program “BEBİS” (version 9.0) [42]. The percentages of energy and nutrient intake for children were calculated by taking into account the age- and gender-specific requirements in the Turkish Nutrition Guide (TÜBER-2022) [43].

### 2.5. The Children’s Eating Behavior Scale

The Children’s Eating Behavior Scale (CEBS), developed by Wardle et al. [44] and validated in Turkish by Yılmaz et al. [45], was used to assess children’s attitudes toward food. This validated Turkish version of CEBS has 35 items, and each item is evaluated on a 5-point Likert scale (never to always). The subscales are grouped into eight categories: voracious eating, emotional overeating, food enjoyment, drinking desire, satiety desire, slow eating, emotional undereating, and food pickiness. The voracious eating subgroup reflects the child’s appetite and inclination towards food; the emotional undereating subgroup reflects the child’s food intake, which varies depending on their mood; the food enjoyment subgroup reflects the child’s interest in food; the drinking desire subgroup reflects the child’s need for beverages; the satiety desire subgroup reflects the child’s tendency to stop eating when they begin to feel full; the slow eating subgroup reflects the child’s tendency to eat slowly; and the food pickiness subgroup reflects the child’s selective eating behavior. The total CEBS score was calculated by averaging the scores of 35 items. Subscale scores were calculated by averaging the total scores of the associated items. Higher subgroup scores on this scale indicate that the nutritional behavior represented by the subgroup is more prevalent. The scale demonstrated good reliability (Cronbach’s α = 0.73) in our study population.

### 2.6. Statistical Analysis

We analyzed the data using IBM SPSS 25.0 (Statistical Package for Social Sciences). Descriptive statistical methods were used in the evaluation of the data as mean ± standard deviation (*X* ± SD), with number and percentage values. The dependent variable was dichotomized according to the presence or absence of sensory integration disorder. The independent variables were age, gender, anthropometric measurements, and the score on the eating behavior questionnaire. The distribution of continuous variables was assessed using the Shapiro–Wilk test and Q–Q plots; histograms were also used for visual inspection. Homogeneity of variances for group comparisons was tested using Levene’s test. To examine differences between groups, one-way analysis of variance (ANOVA) was used for variables that conformed to a normal distribution, whereas the Kruskal–Wallis test was used for variables that did not. The non-parametric Mann–Whitney U test was used to compare the medians of the subgroups. To compare the eating behavior questionnaire scores in autistic children with and without sensory integration disorder, the Pearson correlation test was used for data that were normally distributed, and the Spearman correlation test was used for data that were not normally distributed. Multivariate binary logistic regression was applied to identify independent determinants that distinguish between ASD-only and ASD + SID groups. The initial multivariate model included food intake variables (calcium, iron, vitamin E, magnesium, potassium, vitamin B6, energy, fiber, and polyunsaturated fatty acids) and eating behavior sub-dimensions (skipping meals, enjoying food, ‘craving for beverages’), all of which were found to be significant in univariate analyses. The final multivariate model was derived by backward stepwise elimination, removing variables that did not contribute significantly. VIF/tolerance were used to assess multicollinearity; Hosmer–Lemeshow and AUC-ROC were used to assess model fit and discrimination; and Nagelkerke R^2^ was used for calibration/explanatory power. Age (in years), which differed significantly between groups, was included in the model as a potential confounder. For hypothesis testing, α was set at 0.05; accordingly, confidence intervals were set at 95%, and significance was evaluated at the *p* < 0.05 level.

## 3. Results

### 3.1. Sociodemographic Characteristics

This study involved 36 children with ASD-only and 36 children with ASD + SID, aged 6–15 years. Table 1 summarizes the demographic characteristics of children with ASD-only and those with ASD + SID. Accordingly, the mean age of children with ASD only was significantly lower than that of children with ASD + SID (7.9 ± 2.10 years; 8.8 ± 2.22 years, respectively). The percentage of males was higher in both groups. The percentage of first-born children was higher in the ASD-only group, whereas second-born children were more common in the ASD + SID group (ASD: first child 44.8% vs. second child 38.9%; ASD + SID: first child 33.3% vs. second child 52.8%). There were no statistically significant differences between the groups in gender, age group, birth order, or family history of ASD (*p* > 0.05). The rate of consanguineous marriage in the ASD + SID group was significantly higher than that in the ASD-only group (ASD + SID: 72.2%; ASD only: 58.3%; *p* < 0.05).

### 3.2. Results of Anthropometric Measurements

Table 2 summarizes the anthropometric measurements of children with ASD only and children with ASD + SID. No statistically significant differences were observed in weight, height, WAZ, HAZ, BAZ, or BMI z-scores among the groups (*p* > 0.05). The BMI categories in Z-scores were significantly higher in the female group than in the male group (χ^2^ = 2.93, *p* < 0.05).

### 3.3. Eating Habits and Dietary Intake of Children

Table 3 summarizes the eating habits of children with ASD only and children with ASD + SID. The frequency of main meal consumption differed between groups, but this difference was not statistically significant (*p* > 0.05). However, skipping main meals was more common in the ASD + SID group than in the ASD-only group (97.2% vs. 77.8%; *p* < 0.05). No significant differences were observed in the types of meals skipped (breakfast, lunch, dinner, or snacks; *p* > 0.05).

The mean daily nutrient intakes and nutrient adequacy obtained from the analysis of three-day food consumption records are summarized in Table 4. Children with ASD + SID had significantly lower energy and macronutrient intakes than the ASD-only group. Similarly, intake of fiber, PUFA, and several micronutrients (vitamin E, B1, B6, folate, potassium, calcium, magnesium, phosphorus, and iron) was lower in the ASD + SID group (*p* < 0.05). No significant differences were found in the intake of dietary cholesterol, vitamin A, carotene, vitamin C, sodium, or zinc among groups (*p* > 0.05).

Statistically significant differences in adequacy status for macronutrients, fiber, calcium, iron, vitamin E, magnesium, potassium, and vitamin B6 were found between the groups (*p* < 0.05). In contrast, no statistically significant differences were found between the groups in the adequacy status of vitamin B1, vitamin B2, vitamin C, vitamin A, zinc, folate, and phosphorus (*p* > 0.05).

To assess whether multicollinearity existed among dietary nutrient intake requirement variables, we examined the correlation matrix (Table 5). High correlations in the expected direction and magnitude were observed between macronutrients and energy intake; however, VIF and tolerance were used to evaluate multicollinearity. Based on multiple assessments of linearity, energy uptake was retained in the final model.

The mean scale scores of the children are summarized in Table 6. Accordingly, the scores of children with ASD + SID for the subscale titled “enjoying food” were statistically significantly higher than those of children with ASD only (15.5 ± 3.19 and 13.6 ± 3.06, respectively; *p* < 0.05). The “drinking passion score” subscale was higher in children with ASD + SID than in children with ASD only (*p* < 0.05).

Table 7 indicates the nutritional and eating behavior variables that distinguish the ASD-only and ASD + SID groups. Backward stepwise elimination was used in the multivariate logistic regression analysis. All variables that were found to be significant in relation to nutrient intake and eating behaviors were included in the initial model. In this context, dietary intake of calcium, iron, vitamin E, magnesium, potassium, vitamin B6, energy, fiber, and polyunsaturated fatty acids, skipping meals, and mean scores of the enjoyment-of-food and drinking-passion variables were added to the model. Using the backward elimination method, variables that did not contribute significantly to the model were removed one by one, and the final model included iron intake, potassium intake, and drinking passion. In children with adequate potassium intake, the likelihood of belonging to the ASD + SID group was approximately 92% lower than in those with inadequate potassium intake, suggesting a protective effect of potassium (OR = 0.078). Among behavioral factors, a passion for drinking was also a strong predictor (OR = 2.146, 95% CI [1.365–3.732], *p* < 0.05). Individuals with high drinking passion are approximately 2.1 times more likely to be classified in the ASD + SID group than those with low drinking passion. Folate (µg) was not a significant predictor (*p* > 0.05). The model correctly classified 76.3% of cases and explained approximately 46% of the variance (Nagelkerke R^2^ = 0.46).

## 4. Discussion

This study evaluated the relationships among eating behaviors, dietary nutrient intake, and anthropometric measurements in the ASD-only and ASD + SID groups. We found that children with ASD + SID skip meals more frequently, have a greater need for beverages, and have lower energy and nutrient intakes compared to their peers with only ASD. In our study, the average age of children with ASD only was lower than the average age of children with ASD + SID (7.9 ± 2.10 years vs. 8.8 ± 2.22 years, respectively, *p* < 0.05). The age at diagnosis of ASD varies between 38 and 120 months in childhood [46]. In a meta-analysis, the mean age at diagnosis of ASD was 60.48 months [47]. The heritability of ASD is high, varying between 50% and 90% [48]. In another study, the risk of ASD was 10 times higher in those with first-degree relatives and 2 times higher in those with second-degree relatives [49]. This study found that the consanguinity rate among the parents of children diagnosed with ASD + SID was higher than that among parents of children diagnosed with ASD only (*p* < 0.05).

Children with ASD are at higher risk of being overweight or obese than typically developing children. Obesity in children with ASD can lead to various health problems [50]. In a study of 65 children with ASD and 82 typically developing children, 23.0% were classified as overweight [51]. In studies of children with ASD, the rate of overweight ranges from 30% to 42% [52,53]. This study also found that children with ASD were more likely to be overweight. However, the difference between the groups was not statistically significant. Although anthropometric measurements of children with ASD + SID do not show significant differences compared to children with ASD only, the subsequently observed inadequate food intake highlights the need to evaluate dietary intake. However, assessing nutritional status in children with ASD solely based on anthropometric indicators is insufficient, as this approach risks overlooking subclinical nutritional deficiencies. Therefore, it is recommended that anthropometric data be supported by detailed dietary intake assessments and, where appropriate, biochemical markers. With this approach, our results show directional agreement with the literature, while the effect size is more moderate due to sample and methodological differences.

Feeding problems are common in children with ASD. The most common feeding problems include constantly choosing the same food, food selectivity, food refusal, food neophobia, difficulty chewing, and skipping meals. Such behaviors observed in individuals with ASD during the feeding process may be due to both sensory sensitivities and habits [54,55,56]. A study found that 30% of children with ASD skipped meals, and the most frequently skipped meal was breakfast [57]. In this study, more than half of the children in both groups consumed two main meals and mostly skipped snacks. Children with ASD + SID are more likely to skip meals than children with ASD only, reporting that reasons for skipping meals included lack of appetite and habitual behaviors. This pattern is directionally consistent with findings in the literature on food selectivity, food refusal, and habitual restrictions reported in OCD, all of which can disrupt meal structure. However, specific details—for example, that breakfast is the most frequently skipped meal—are sensitive to contextual factors, including sample age distribution, cultural mealtimes, and school and home routines, which results in variability in frequency and effect size among different studies. In our study, although family reports indicate anorexia and habitual behaviors as the primary justification, the presence of accompanying sensory processing difficulties (OSD + SID) emerges as a possible mechanism that increases the frequency of these behaviors. In other words, our findings support the hypothesis that sensory profile may have a multiplicative or reinforcing effect on meal structure.

Eating behavior problems are common in children with ASD [26]. In a study, 55.70% of individuals reported a fear of tasting new foods, 55.70% reported not being open to new tastes, 54.43% reported food selectivity, and 32.91% reported beverage selectivity [58]. In a study that used the eating behavior scale in children, children with ASD exhibited greater emotional overeating than typically developing children [59]. When similar scale scores were evaluated in this study, it was determined that the food enjoyment subscore of children with ASD + SID was statistically significantly higher than that of children with ASD only (15.5 ± 3.19 and 13.6 ± 3.06, respectively; *p* < 0.05). In addition, children with ASD + SID had higher drinking passion subscale scores than children with ASD only (*p* < 0.05). The total scale score of children with ASD + SID was higher than that of children with ASD-only. The results of our study are directionally consistent with findings that eating behavior problems (food pickiness, avoidance of new tastes, food/drink-based restrictions) reported in ODD are common. However, our study suggests that the relative weight of hedonic/desire-based dimensions (pleasure, beverage cravings) may be increased in children with accompanying sensory processing difficulties. While some studies in the literature report high rates of emotional overeating or neophobia, effect sizes and subscales are sensitive to factors such as age range, scale/version used, scope of parental report, cultural beverage/snack patterns, and sample selection.

Sensory integration disorder and sensory hyper/hyposensitivity can manifest across various sensory modalities, including touch, smell, taste, hearing, vision, and movement. Sensory integration problems are common in ASD, with reported incidence rates of 65–90% [60]. Sensory integration problems related to nutrition include difficulties with the smell, taste, temperature, and texture of foods [61]. Autism is caused by differences in sensory processing (hearing, smell, taste, touch, and vision), leading the brain to over- or under-regulate sensory information, which causes children to respond hyper- or hypoactively to environmental stimuli [62]. Eating problems are common in children with ASD and are associated with sensory selectivity and a tendency to reject new foods [23,63]. A study of 1443 autistic children reported atypical eating behaviors in 70.4% of children; approximately 80% of these children had difficulties with sensory processing or modulation, suggesting that food selectivity stems from these sensory differences. Children with ASD often prefer high-energy, low-quality foods. This tendency can lead to deficiencies in certain micronutrients. Vitamin and mineral deficiencies are particularly common [64]. Similarly, in a study of 65 Spanish and 62 Colombian children with ASD, Colombian children showed greater sensory responsiveness than Spanish children [13]. Our findings suggest that a more sensitive assessment focusing on diversity and micro-nutrient adequacy is needed rather than generalizations such as a “shift towards high-energy, low-quality foods”. Therefore, when interpreting eating problems in ASD, a context-sensitive examination of the relationship between sensory profile (hyper- and hyporeactivity, integration difficulties) and eating patterns (variety, neophobia, beverage and snack preferences) may be more clinically informative.

Nutritional assessment is the first step in identifying nutritional problems and initiating appropriate intervention [65]. Data from different countries show significant differences in levels of certain nutrients and antioxidants when comparing children with ASD to their typically developing peers [66,67,68]. In this study, total energy and macronutrient intakes were significantly lower in the ASD + SID group than in the ASD-only group. Similarly, a lower dietary intake of fiber, PUFAs, and micronutrients (vitamin E, B1, B6, folate, potassium, calcium, magnesium, phosphorus, and iron) was observed in the ASD + SID group (*p* < 0.05). This result indicates the hypothesis that sensory integration difficulties may have a negative and selective effect on food quality and adequacy. In contrast, no significant differences were observed between the groups in dietary intakes of cholesterol, vitamin A, carotene, vitamin C, sodium, and zinc (*p* > 0.05). In a study of 33 school-aged autistic children assessing the relationship between nutrients in one-carbon metabolism and sensory processing, dietary vitamin B12 intake was negatively correlated with oral and sensory field scores, whereas vitamin B1 was positively correlated with visual field scores. Furthermore, unlike other studies, a study reported that more than 80% of children with ASD met their respective DRIs for vitamin B1, vitamin B2, vitamin B5 (pantothenic acid), vitamin B6, folate, vitamin B12, vitamin C, iron, zinc, and magnesium [61]. A study comparing children with ASD and typically developing preschoolers found that the ASD group consumed more energy-dense foods and fewer fruits and vegetables than their typically developing peers [51]. A study investigating the relationship between sensory hypersensitivity and food selectivity that involved 32 children with ASD and 25 neurotypical children found that children with ASD had significantly higher levels of sensory hypersensitivity. Furthermore, it was reported that children with ASD and sensory hypersensitivity had lower dietary intake of vegetables, dairy products, animal protein, and legumes compared to neurotypical children [10]. In line with these studies, this pattern suggests that sensory processing difficulties may affect dietary intake and nutrient requirement ratios—particularly for certain micronutrients and PUFAs—through food selectivity and a narrowed food repertoire, and that some other nutrients (e.g., sodium, cholesterol) may be supplied by conventional or processed foods. These findings support the prioritization of individualized interventions in the nutritional management of the ASD + SID subgroup, such as sensory-based behavioral strategies to increase food diversity, targeted micronutrient supplementation, and PUFA enrichment. Applied behavior analysis (ABA)-based interventions in challenging eating situations for children with ASD have been reported to increase food acceptance and reduce compulsive mealtime behaviors [69].

## 5. Conclusions

This study compared dietary intake and eating behaviors between children with ASD only and those with ASD + SID. In our study, the rate of consanguineous marriage was higher among parents of children with ASD + SID. The children with ASD + SID skipped meals more frequently, and had a greater interest in food and a greater need for beverage consumption. Furthermore, children with ASD + SID had lower energy and nutrient intakes than children with ASD only. Preferring low-nutrient, energy-dense foods can alter their metabolism, promote the accumulation of oxidative radicals, and cause mental and physical deterioration. Raising awareness of diet, nutrition, and obesity is difficult among children with special needs. Despite their efforts, parents of such children are often unable to help control their children’s eating because tantrums and behavioral problems are common. Families need comprehensive support systems and face difficulties in providing appropriate care for their children [70]. Therefore, it may be recommended that doctors and parents collaborate with nutritionists and dietitians to help these children maintain physical fitness and improve their quality of life by eating a healthy diet. Furthermore, educational services for children with ASD only and children with SID should be integrated into the healthcare system and social policies. In addition to providing families with nutritional information, it is recommended that these families convene periodically to raise public awareness and foster social cohesion. These findings indicate that dietary intake is more negatively affected in the ASD + SID subgroup in terms of both quantity (energy/macronutrients) and quality (fiber, PUFA, selected micronutrients). Therefore, in addition to anthropometry, structured dietary intake assessment (preferably recording 2 weekdays + 1 weekend day) and, if necessary, biochemical markers (e.g., ferritin, 25(OH)D, B12, folate) should be routinely implemented during follow-up. Clinical management requires the coordination of individualized interventions such as sensory-based behavioral strategies (increasing food acceptance) and targeted micronutrient supplementation. A multidisciplinary approach and care coordination (pediatrician/psychiatrist–dietitian–psychologist) is recommended, taking into account the family’s consanguinity history and parental burden.

There are several limitations to the present study. The cross-sectional design of the study does not allow for establishing cause-and-effect relationships. Second, the sample size was relatively small, and the sample was selected from a single region; this may limit the generalizability of the results. Therefore, this research is a pilot study due to the limited sample size. Finally, the children included in the study ranged in age from 6 to 15 years, creating a developmentally heterogeneous group. It should be considered that sensory processing characteristics, eating behaviors, and nutritional habits can differ significantly between childhood and adolescence. Future studies conducted with narrower age ranges and larger sample sizes will increase the validity and generalizability of the results.

Future studies should implement longitudinal dietary intervention programs that consider sensory integration difficulties and monitor the effects of these interventions on nutrient intake, growth indicators, and eating behaviors over time. Evaluating the effectiveness of nutritional strategies tailored to sensory characteristics will contribute to the development of more targeted nutritional approaches in the ASD-only and ASD + SID groups.

## Figures and Tables

**Table 1:** Sociodemographic characteristics of children.

General Characteristics	ASD (*n* = 36)		ASD + SID (*n* = 36)		*p* Value
	*n*	%	*n*	%	
Gender					
Female	15	41.7	11	30.6	0.326 ^a^
Male	21	58.3	25	69.4	
Age (years)					
6–9	29	80.6	26	72.2	0.405 ^a^
10–14	7	19.4	10	27.8	
(Mean ± SD)	7.9 ± 2.10		8.8 ± 2.22		0.043 ^b^
Birth order					
1	16	44.4	12	33.3	0.374 ^c^
2	14	38.9	19	52.8	
≥3	6	16.7	5	13.9	
Family history with ASD					
Yes	3	8.3	2	5.6	0.500 ^d^
No	33	91.7	34	94.4	
Relationship status between parents					
Yes	15	41.7	26	72.2	0.009 ^a^
No	21	58.3	10	27.8	

ASD: Autism Spectrum Disorder; ASD + SID: Autism Spectrum Disorder with Sensory Integration Dysfunction; SD: standard deviation; ^a^ Pearson Chi-Square; ^b^ Mann–Whitney U test; ^c^ likelihood ratio; ^d^ Fisher’s exact test. *p* < 0.05 was considered statistically significant.

**Table 2:** Anthropometric measurements of children.

Measurements	Female		Male		*p*_1_ Value	*p*_2_ Value	*p*_3_ Value
	ASD (Mean ± SD)	ASD + SID (Mean ± SD)	ASD (Mean ± SD)	ASD + SID (Mean ± SD)			
Weight for age (percentile)	64.5 ± 37.77	74.5 ± 30.59	56.6 ± 37.39	48.8 ± 35.06	0.951	0.342	0.710
Height for age (percentile)	78.8 ± 28.45	78.0 ± 30.51	67.7 ± 09.03	61.7 ± 26.54	0.262	0.372	0.404
WAZ	1.2 ± 1.37	1.1 ± 0.68	0.8 ± 1.50	0.5 ± 1.22	0.828	0.404	0.336
HAZ	2.2 ± 1.88	1.3 ± 0.91	1.1 ± 1.35	0.7 ± 1.25	0.281	0.354	0.137
BAZ	0.2 ± 1.19	0.6 ± 0.78	0.3 ± 2.27	−0.1 ± 1.43	0.259	0.998	0.733
**BMI Group in Z Score**	**ASD** **(*n* = 36)**		**ASD + SID** **(*n* = 36)**				
	***n***	**%**	***n***	**%**			
≤−2 (Z < −2)	7	19.4	6	16.7	-	-	0.570
−2 to −1	6	16.7	5	13.9			
−1 to +1	11	30.6	17	47.2			
+1 to +2	11	30.6	8	22.2			
≥+2 (Z > +2)	1	2.7	-	-			

ASD: Autism Spectrum Disorder; ASD + SID: Autism Spectrum Disorder with Sensory Integration Dysfunction, WAZ: Weight-For-Age Z-score; HAZ: Height-For-Age Z-score, BAZ: BMI-For-Age Z-score, BMI: body mass index. Likelihood ratio. The *p*_1_ value indicates the significance of the difference between the means of the girls’ anthropometric measurements. The *p*_2_ value indicates the significance of the difference between the means of anthropometric measurements of boys. The *p*_3_ value indicates the significance of the difference between the means of anthropometric measurements of boys and girls.

**Table 3:** Eating habits of children.

General Characteristics	ASD (*n* = 36)		ASD + SID (*n* = 36)		*p* Value
	*n*	%	*n*	%	
Number of main meals					
1	2	5.6	2	5.6	0.596 ^a^
2	20	55.6	24	66.7	
3	14	38.8	10	27.7	
Skipping main meal					
Yes	28	77.8	35	97.2	0.014 ^a^
No	8	22.2	1	2.8	
Skipped main meal					
Breakfast	5	17.2	1	2.9	0.165 ^a^
Lunch	5	17.2	11	31.4	
Dinner	2	6.9	3	8.6	
Snacks	17	58.6	20	57.1	

ASD: Autism Spectrum Disorder; ASD + SID: Autism Spectrum Disorder with Sensory Integration Dysfunction. ^a^ Pearson Chi-Square; *p* < 0.05 was considered statistically significant.

**Table 4:** Dietary nutrient intake and adequacy (% of RDA) of children.

Energy and Nutrients	ASD (*n* = 36) ($\bar{X}$ ± SD)	ASD + SID (*n* = 36) ($\bar{X}$ ± SD)	Meeting the Requirements (%)		*p*_1_ Value	*p*_2_ Value
			ASD (*n* = 36)	ASD + SID (*n* = 36)		
Energy (kcal)	868.9 ± 283.0	693.9 ± 125.8	49.7 ± 17.65	37.8 ± 7.00	0.001	<0.001
Protein (g)	32.8 ± 12.2	26.1 ± 6.6	121.2 ± 55.0	85.0 ± 25.6	0.005	0.001
Carbohydrates (g)	93.4 ± 35.1	73.9 ± 16.6	71.8 ± 27.0	56.8 ± 12.7	0.004	0.004
Fat (g)	39.6 ± 13.9	32.0 ± 7.7	72.9 ± 27.76	56.2 ± 14.03	0.006	0.002
Fiber (g)	7.4 ± 3.7	5.7 ± 2.3	46.7 ± 23.07	34.9 ± 13.58	0.022	0.010
Cholesterol (mg)	223.0 ± 119.9	193.1 ± 103.6	-	-	0.261	-
Vitamin A (µg)	472.5 ± 301.0	378.2 ± 124.9	141.4 ± 96.02	111.8 ± 40.01	0.087	0.092
Vitamin E (mg)	5.9 ± 3.6	4.0 ± 1.6	62.6 ± 40.90	40.2 ± 13.78	0.005	0.003
Vitamin B1 (mg)	0.41 ± 0.16	0.34 ± 0.10	136.1 ± 52.43	114.2 ± 34.07	0.039	0.039
Vitamin B2 (mg)	0.82 ± 0.34	0.69 ± 0.20	99.1 ± 42.92	81.7 ± 22.40	0.050	0.035
Vitamin B6 (mg)	0.59 ± 0.28	0.40 ± 0.15	62.3 ± 29.33	39.9 ± 15.86	0.001	<0.001
Folate (µg)	128.8 ± 55.2	105.9 ± 29.9	78.1 ± 35.04	63.1 ± 17.18	0.032	0.023
Vitamin C (mg)	39.8 ± 17.7	33.0 ± 16.9	93.5 ± 38.14	78.1 ± 42.35	0.098	0.108
Sodium (mg)	2461.0 ± 2824.7	1671.3 ± 1058.5	142.6 ± 166.54	96.1 ± 62.62	0.121	0.121
Potassium (mg)	1126.1 ± 426.6	811.3 ± 187.3	68.9 ± 30.73	46.4 ± 16.05	<0.001	<0.001
Calcium (mg)	457.3 ± 204.8	375.4 ± 131.2	65.1 ± 31.03	52.2 ± 17.9	0.047	0.034
Magnesium (mg)	124.1 ± 48.2	97.7 ± 25.1	52.3 ± 21.45	39.9 ± 9.61	0.005	0.002
Phosphorus (mg)	604.5 ± 215.5	493.3 ± 119.3	132.4 ± 50.90	105.6 ± 25.22	0.008	0.006
Iron (mg)	4.41 ± 1.25	3.66 ± 0.90	55.6 ± 16.8	45.9 ± 11.34	0.004	0.006
Zinc (mg)	4.47 ± 1.70	4.00 ± 0.85	69.6 ± 28.38	61.0 ± 13.70	0.139	0.106

ASD: Autism Spectrum Disorder; ASD + SID: Autism Spectrum Disorder with Sensory Integration Dysfunction. $\bar{X}$: mean, SD: standard deviation. Student’s *t*-test: a *p*_1_ value indicates the significance of the difference between the means of dietary nutrient intake, and a *p*_2_ value indicates the significance of the difference between the means of nutrient adequacy. *p* < 0.05 was considered statistically significant.

**Table 5:** Correlation of daily energy and nutrient intake percentages between groups.

Variables	Energy (kcal)	Protein (g)	Carbohydrates (g)	Fat (g)	Fiber (g)	PUFA (g)	Cholesterol (mg)	Group
Energy (kcal)	—	0.83	0.89	0.90	0.75	0.66	0.36	−0.47
Protein (g)		—	0.61	0.76	0.59	0.35	0.64	−0.40
Carbohydrates (g)			—	0.62	0.73	0.54	0.14	−0.42
Fat (g)				—	0.60	0.73	0.37	−0.40
Fiber (g)					—	0.57	0.03	−0.33
PUFA (g)						—	0–0.03	−0.30
Cholesterol (mg)							—	−0.17
Group (0 = ASD, 1 = ASD + SID)								—

ASD: Autism Spectrum Disorder; ASD + SID: Autism Spectrum Disorder with Sensory Integration Dysfunction.

**Table 6:** Distribution of Children’s Eating Behavior Scale scores.

Children’s Eating Behavior Scale	ASD (*n* = 36) ($\bar{X}\;$ ± SD)	ASD + SID (*n* = 36) ($\bar{X}\;$± SD)	*p* Value
Subscale scores of CEBS			
Eager to eat	15.2 ± 2.48	15.5 ± 1.80	0.516
Emotional overeating	10.9 ± 1.57	11.6 ± 1.66	0.073
Enjoying food	13.6 ± 3.06	15.5 ± 3.19	0.011
Drinking passion	9.0 ± 2.02	10.2 ± 1.42	0.005
Fullness eagerness	20.0 ± 2.14	20.4 ± 2.19	0.482
Slow eating	11.8 ± 2.29	11.2 ± 2.18	0.321
Emotional undereating	12.9 ± 2.21	13.2 ± 1.59	0.584
Food pickiness	9.9 ± 1.17	10.1 ± 1.05	0.825
Total CEBS score	103.3 ± 10.53	107.7 ± 7.09	0.040

ASD: Autism Spectrum Disorder; ASD + SID: Autism Spectrum Disorder with Sensory Integration Dysfunction, CEBS: Children’s Eating Behavior Scale. $\bar{X}$: mean, SD: standard deviation. Student’s *t*-test: *p* < 0.05 was considered statistically significant.

**Table 7:** Multivariate logistic regression models.

Variables	OR (95% CI)	*p* Value
Folate (µg)	0.23 (0.026–1.587)	0.155
Potassium (mg)	0.08 (0.012–0.348)	0.002
Drinking passion	2.15 (1.365–3.732)	0.003

OR: Odds ratio; *p* < 0.05 was considered statistically significant.

## Data Availability

The data that support the findings of this study are available from the first author upon reasonable request. The data are not publicly available due to [a commitment to protect the confidentiality of the research data was included in the ethics committee application].

## Abbreviations

The following abbreviations are used in this manuscript:

ASD	Autism Spectrum Disorder
BAZ	BMI-For-Age Z-score
BMI	Body mass index
CDC	Centers for Disease Control and Prevention
CEBS	Children’s Eating Behavior Scale
HAZ	Height-For-Age Z-score
SD	Standard deviation
SID	Sensory integration dysfunctions
WAZ	Weight-For-Age Z-score
WHO	World Health Organization
